# Detection of human vital signs in hazardous environments by means of video magnification

**DOI:** 10.1371/journal.pone.0195290

**Published:** 2018-04-11

**Authors:** Celestino Ordóñez, Carlos Cabo, Agustín Menéndez, Antonio Bello

**Affiliations:** 1 Department of Mining Engineering, Geomatics and Computer Graphics Research Group, Universidad de Oviedo, Mieres, Asturias, Spain; 2 Department of Manufacturing Engineering, Universidad de Oviedo, Gijón, Asturias, Spain; Case Western Reserve University Jack Joseph and Morton Mandel School of Applied Social Sciences, UNITED STATES

## Abstract

In cases of natural disasters, epidemics or even in dangerous situations like an act of terrorism, battle fields, a shooting or a mountain accident, finding survivors is a challenge. In these kind of situations it is sometimes critical to know if a person has vital signs or not, without the need to be in contact with the victim, thus avoiding jeopardizing the lives of the rescue workers. In this work, we propose the use of video magnification techniques to detect small movements in human bodies due to breathing that are invisible to the naked eye. Two different video magnification techniques, intensity-based and phase-based, were tested. The utility of these techniques to detect people who are alive but injured in risk situations was verified by simulating a scene with three people involved in an accident. Several factors such as camera stability, distance to the object, light conditions, magnification factor or computing time were analyzed. The results obtained were quite positive for both techniques, intensity-based method proving more adequate if the interest is in almost instant results whereas the phase-based method is more appropriate if processing time is not so relevant but the degree of magnification without excessive image noise.

## Introduction

In recent years, there have been many advances in computer science that have made possible the creation of novel systems with application in different fields such as medicine, archeology, industry or video games, inter alia. For example, in forensic medicine computer vision and close-range photogrammetry procedures allow to reconstruct the scene of a crime. In this context, some 3D image reconstruction systems have been recently developed for specific use in forensic medicine. They allow the non-contamination of the crime-secene, the reduction of data collection time, and the lack of contact with the victims. A system composed of a 3D laser scanner, a profilometer and a low-cost digital camera for crime scene documentation was proposed in [1]. Its developers concluded that this kind of technologies offer an objective multi-resolution database, very useful for evidence analysis, performing police investigation or supporting legal medical studies. Another approach [2] consisted of a system using open source tools for the automatic computation of a virtual 3D scene, combining photogrammetric and computer vision algorithms, like ASIFT (Scale Invariant Affine Transform) and SGM (semi-global matching algorithm).

The introduction of artificial intelligence (AI) also constitutes a major advance in technologies applied to emergency response and forensic medicine. In [3], an artificial neural network model was used to construct an environmental emergency decision support system. The system was illustrated with a example of an unexpected atmospheric accident in a district of Shanghai. In [4], the authors reported the construction of a robot for human detection, specially designed for natural disasters. The robot was mobile, and it was equipped with a sensor to detect the victims, an arm to remove the obstacles, a camera to capture images that are sent to a control unit, and a microcontroller (a programmable integrated circuit capable of executing commands recorded in its memory), which was the core of the system. The different sensors monitored current readings and sent data to the microcontroller in real time. In addition, the robot used the camera to detect motion using computer vision algorithms. Similar robots were proposed in [5] and [6], both based on sensors that capture data, microcontrollers, computer vision algorithms and AI.

Another important contribution of the technology in medicine, including AI, is the development of solutions to help people with disabilities. There are a large number of works based on communication technologies, computing, digital electronics and signal processing for human vital sign monitoring, including their detection or measurement remotely [7]. The most relevant steps in these methodologies are: the incorporation of some components or sensors for the data acquisition, the transmission of data from patient to hospital, the victim response, the associated doctor’s decision, and the storage of all the data. Authors in [8] created a system for people confined to bed at home but who are able to move their head, lips and eyebrows intentionally. This system incorporated a vision input device which uses slight movements of the mentioned body parts to communicate the patient with other people, such as relatives or friends. This input device could be adapted according to the user’s symptoms. A PC controlled robot with a PIR sensor (passive infrared sensor) that measured infrared light invisible to the human eye was described in [9]. When motion was detected by this sensor, the robot transmitted the information through a microcontroller that sent a message using a GSM (Global System for Mobile Communications) based wireless modem that included the geographic coordinates obtained by a GPS receiver. In [10], a study to analyze the benefits and the drawbacks of video technology to improve safety management in manufacturing companies was conducted. The conclusion was that this technology can be very useful for detecting riks in working environments.

In the signal processing field there are also some valuable studies. For instance, a system combining radar applications with advanced signal processing algorithms for the detection, location in search and rescue of trapped victims in dangerous environments [11]. With a similar purpose, a portable Doppler radar system and self-correlation and adaptive line enhancer methods to design a procedure with minimal interference of any moving objects around the victim were developed [12]. A realtime methodology for the continuous monitoring of the vital signs of the victims and tracking their locations was reported in [13]. It was composed of location sensors for indoor and outdoor use, a pulse oximeter, a blood pressure sensor an electronic triage tag. The data collected by the sensors was transmitted through a web portal, so it can be analysed by a medical staff.

There are other works exclusively based on images remotely measured that look for changes in pixel values. For instance, a system that analyzes color variations in the skin tone was proposed in [9]. An analogous system using a basic webcam is described in [14]. Recently, an algorithm, that was implemented on an Android smartphone, was developed to estimate the respiration rate from the video of the smartphone’s camera [15].

In this work we propose a new approach to detect alive people in hazardous environments which has two main characteristics: i) No physical contact with the injured people is necessary, avoiding unnecessary risk to doctors, police or rescue workers, ii) the only measuring device required is a low cost video-camera.

## Methodology

Our goal is to look for a fast and cheap method based on signal processing to reveal vital signs or imperceptible movements to the naked eye in injured people, by just processing a video of the scene. To reveal subtle temporary body movements due to breathing, a non-professional video camera or even the camera of a mobile phone can be used. Motion analysis is carried out using two video image processing techniques: one called Eulerian Linear Video Magnification (ELVM) [16], and other named Phase-Based Video Motion Processing (PBVM) [17]. Both methods are compared and analyzed their advantages and disadvantages.

The idea behind both methods is the combination of spatial and temporal filtering. First, a multi-scale signal representation of the images, named pyramid (Laplacian, Gaussian or Steerable) is carried out. Pyramids are constructed using spatial filters that decompose the frames into different spatial frequency bands in order to obtain, from an input image, another image with more appropriate features for a specific application. Gaussian pyramids are suitable for color magnification while Laplacian and Steerable pyramids are more appropriate for motion magnification. Second, a temporal filtering is applied to the elements of the pyramid showing the motion at specific temporal frequencies selected by the user. This is done in a uniform way for all spatial levels (or bands) and for all pixels within each level.

**Fig 1:**
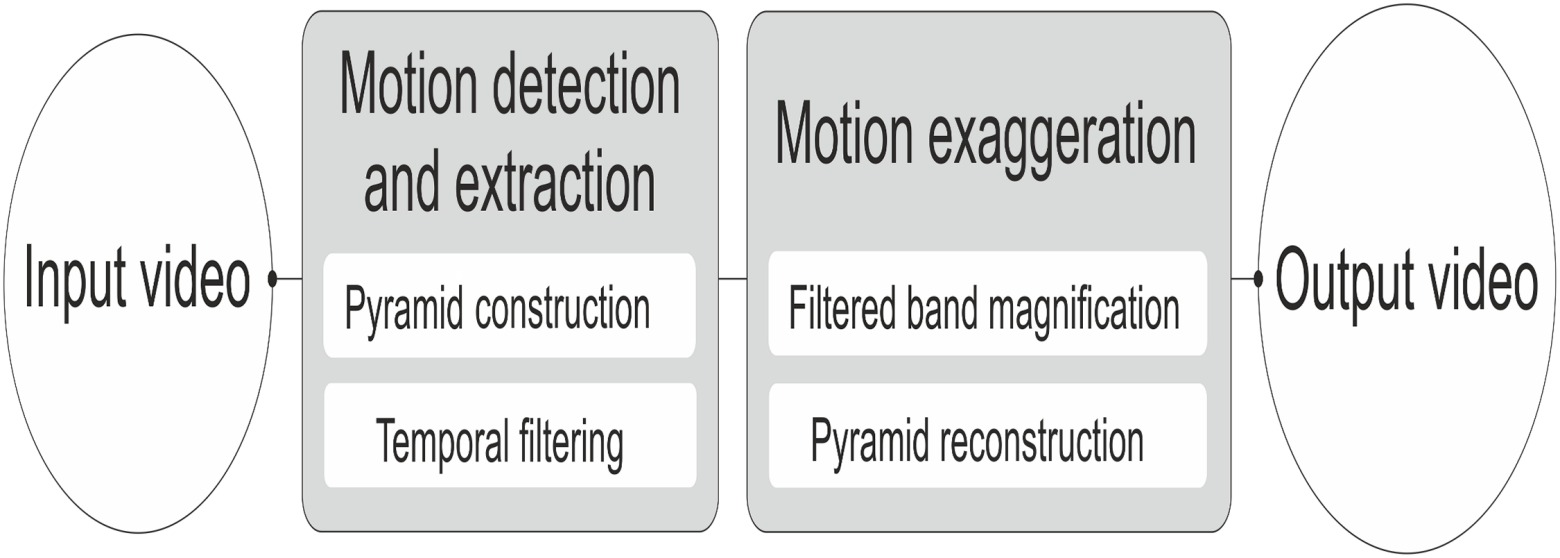
Diagram of the video motion magnification procedure.

One of the main differences between ELVM and PBVM is the type of pyramid used. While ELVM computes a full Laplacian pyramid, PBVM computes the phase variations of a complex-valued Steerable pyramid over time. In both cases the magnification is applied over each level in the pyramid, and not over the original images. This is due to the fact that the goal is to magnify the levels of the pyramid that contain the frequencies of the movement. Finally, the resulting signal is multiplied by a magnification factor *α*, specified by the user, which amplifies the original motion in order to let us appreciate those movements that are imperceptible for the naked eye. Then, the spatial pyramid with the magnified values is collapsed and added back to the original to obtain the final video with the motion exaggerated. These two procedures do not explicitly estimate displacements, but rather exaggerate the motion to reveal hidden information. Fig 1 shows the basic steps in both video motion magnification techniques.

A more detailed explanation of these techniques is provided below.

### Data acquisition

The first step of the proposed method is, of course, to take a video of the scene. The video is recorded with a video camera from a stable position, in order to avoid undesirable noise due to the camera movements. Thus, only movements of the object scene are recorded. In addition, if the area around the victim is considered unsafe for the emergency staff (e.g. rescue workers in a terrorist attack), the camera can be placed on a vehicle or a robot that should be stopped at the time of recording the video. In order to achieve the best results, it is advisable to use an FHD, or even better a 4K, video camera. Furthermore, it is convenient to record images as clear as possible and the camera frame rate must satisfy the Nyquist-Shannon theorem:

$$\begin{array}{c} f_{s}>2\times f \end{array}$$

where *f* is the highest frequency contained in the signal and *f*_*s*_ is the sampling rate (how often samples are taken per unit of time).

### Eulerian linear motion magnification

Before the development of the Eulerian methods, the most common technique for motion exaggeration was the Lagrangian approach. This method focuses on individual points and analyzes their change in location over time. Therefore, it performs a tracking of points over time, so it requires a unique matching method between points or patches. For this reason, Lagragian methods are expensive in terms of computation. In contrast, the goal of the Eulerian approach is to study a certain characteristic at pixel level, such as the intensity, over time. Thus, Lagragian methods estimate where a given point moves to, while Eulerian methods measure flux properties of the scene.

One of the most notable characteristics of the Eulerian approach is that it is based on local pixel-level operations in an efficient computation way, avoiding the need for solving optimization problems, in contrast to the Lagragian methods based on dense optical flow estimation or matching sparse feature points. As a drawback, both are limited to small motions between frames.

According to [16, 17], in the Eulerian motion magnification a multi-scale decomposition is performed on each frame of the input video using Laplacian pyramids (in fact, a pyramid is constructed for each RGB channel). Afterwards, the same temporal filtering is carried out for all pixels in the pyramid for each pair of consecutive frames. The resulting signal is amplified in each spatial frequency band applying a magnifcation factor. Finally, disintegrating the spatial pyramid with the magnified values (following the reverse procedure used to create it) and adding back the magnified signal to the original, it is possible to reconstruct the final output video which contains the amplified movements.

From a mathematical point of view, let us assume that *I*(*x*, *t*) represents a one dimensional image intensity profile that is translated over time. If *I*(*x*, 0) = *f*(*x*), then the intensity at instant *t* can be expressed as *I*(*x*, *t*) = *f*(*x* + *δ*(*t*)), where *δ*(*t*) represents the translation of the signal over time. As the goal is to magnify the movement in an amount *α*, named “magnification factor”, which is selected by the user, obtaining a synthesized signal $\hat{I}\left(x,t\right)=f\left(x+\left(1+\alpha \right)\delta \left(t\right)\right)$.

Applying a first-order Taylor expansion to the previous signal, a relatively simple way of estimating the magnified signal is obtained

$$\begin{array}{c} \hat{I}\left(x,t\right)==I\left(x,0\right)+\left(1+\alpha \right)\delta \left(t\right)\frac{\partial I(x,t)}{\partial x} \end{array}$$ (1)

It consists in adding to the original signal *I*(*x*, 0), (1 + *α*) times the result of applying a temporal bandpass filter to the signal at each instant of time, represented by $\delta \left(t\right)\frac{\partial I(x,t)}{\partial x}$. This expression is only valid for smooth images and small motions. According to [16], there is an upper limit for the parameter *α* given by:

$$\begin{array}{c} \left(1+\alpha \right)\delta \left(t\right)\leq \frac{\lambda }{8} \end{array}$$ (2)

Initially, a representative wavelength (spatial frequency cutoff), λ, of the lowest band in the Laplacian pyramid, which reveals small motions, can be tried. This wavelength λ must provide information about the dimensions of the image at that level. Then, an estimation of this parameter can be obtained by calculating the diagonal length of the image [18].

#### Laplacian pyramids: Spatial filtering over the frames

**Fig 2:**
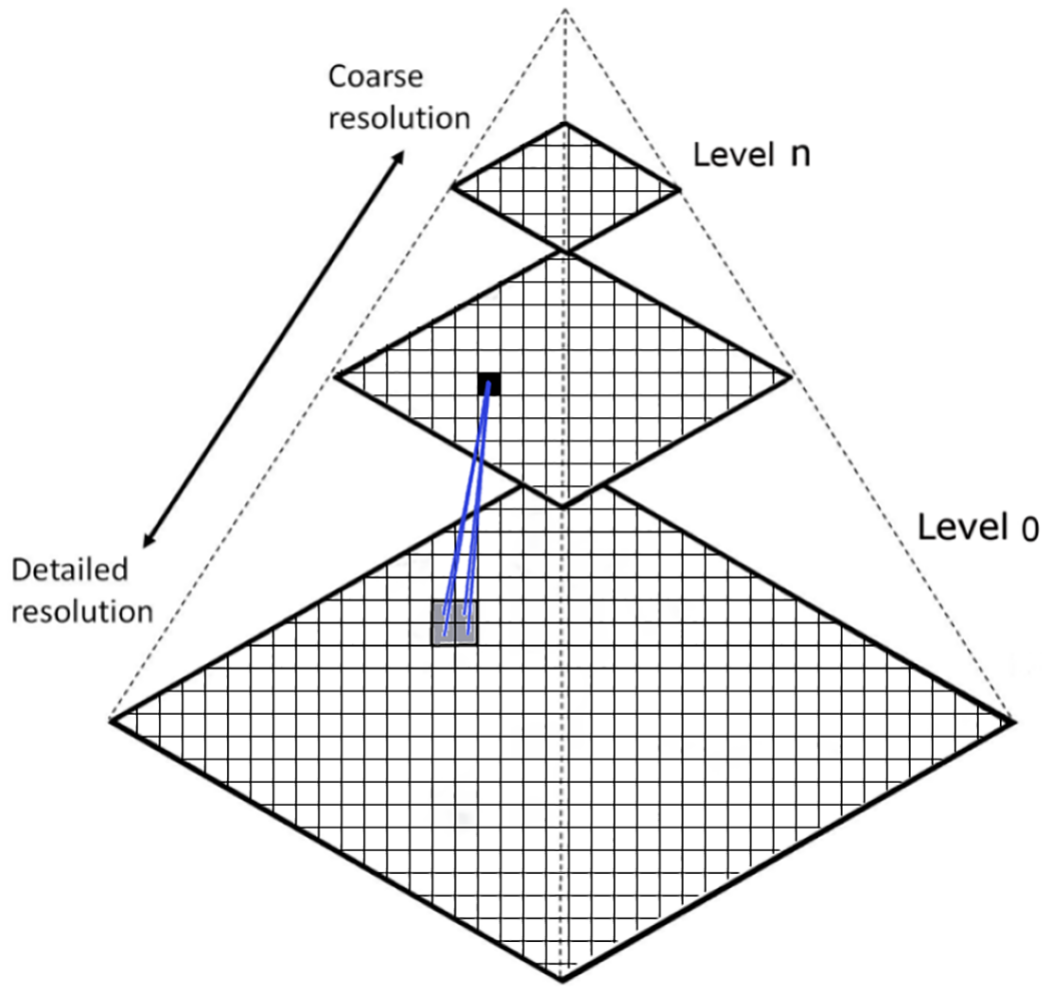
Pyramid representation of an image showing the different levels of resolution.

A Laplacian pyramid is a multi-scale signal representation of an image which is based on recursively applying a lowpass filter and a reduction in size to obtain a series of images with less detail and smaller dimensions. To build it, the difference between the downsampled and the original images at each dimension must be computed. If this pyramid decomposition is applied over an image and then rebuild it, the original image without loss (except for possible rounding errors) is obtained. Fig 2 shows a representation of the pyramid construction.

**Fig 3:**
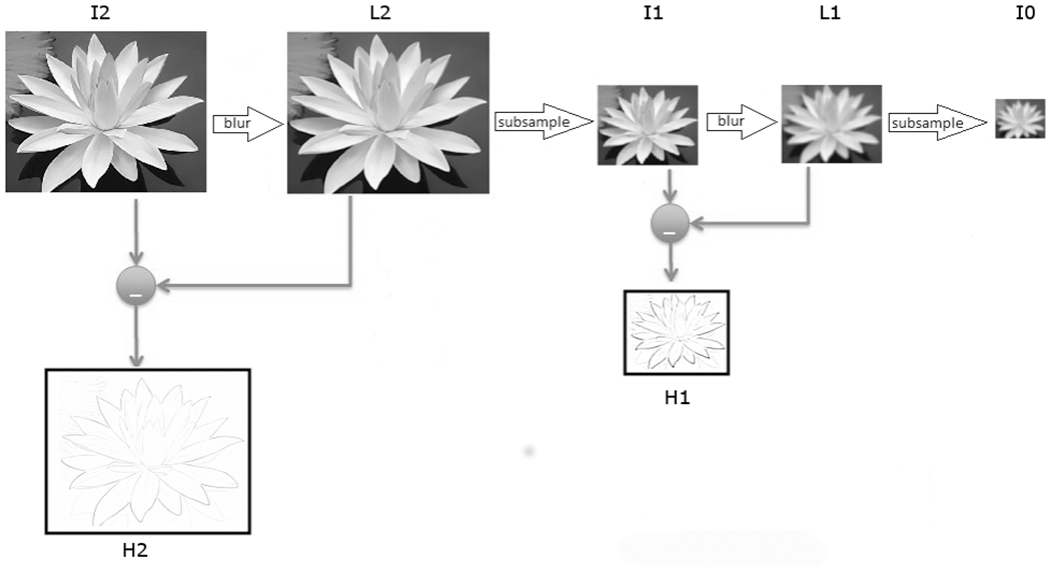
Construction of a Laplacian pyramid with three levels. The first row shows the procedure to build a Gaussian pyramid by successively smoothing (blurring) and downsampling the images. I2, I1 and I0 are elements of the Gaussian pyramid, while H2, H1 and I0 are the elements of the Laplacian pyramid. H2 and H1 are high-pass subbands obtained by subtracting two adjacent images of the Gaussian pyramid.

When the original image is successively smoothed using a Gaussian filter (blur), and scaled down, a Gaussian pyramid is constructed. Accordingly, a Gaussian pyramid is a sequence of low-pass, down-sampled images. A Laplacian pyramid is similar to a Gaussian pyramid; in fact, each image at certain level of the Laplacian pyramid is the difference between two corresponding adjacent levels of the Gaussian pyramid. Only the smallest level is not a difference of two images, allowing in this way to reconstruct the original image from the difference images. Then, a Laplacian pyramid can be considered a sequence of band-pass, down-sampled images. Fig 3 represents schematically the process of construction of a Laplacian pyramid with three levels.

#### Temporal filtering and motion amplification

Once the pyramid for each frame has been constructed, a temporal filter is applied to the temporal series of pixels in each spatial band of the pyramid, in order to extract the frequency bands of interest. The temporal filtering approximates spatial translation of the signal according to Eq (1). The choice of temporal filter is application dependent. In our specific application the best results were obtained through the implementation of a band pass Butterworth filter with cutoff frequencies *f*_*h*_ and *f*_*l*_, where (*f*_*h*_, *f*_*l*_) is the interval that contains the frequencies of motion to magnify. Butterworth filters are characterized by having a magnitude response that is maximally flat in the passband and monotonic overall.

Once each level of the pyramids has been temporary filtered, the amplification factor *α* is applied to each of them. Several values of *α* can be tested but, in order to improve the results, it is advisable to use a constant value for the spatial bands that are within the limits given by Eq (2). For higher frequencies, the magnification factor is attenuated linearly [7]. The result is a set of pyramids with magnified values that will be finally reconstructed using the inverse technique in order to get the final magnified video.

### Phase-based motion magnification

The intensity-based video magnification approach has a notable drawback: the noise increases linearly with the amplification factor, so it supports small amplification factor values. In order to avoid this inconvenience, the phase-based video motion magnification, that is based on the Fourier transform, was developed [18]. It is theoretically somewhat more complex than the Eulerian approach, although it also consists in the application of spatial filters over each frame, but constructing a different type of pyramid: a Steerable pyramid [19]. This decomposition can be considered a version of the Laplacian pyramid for different directions (orientations). The main difference between both methods is the type of filters applied for their creation. The phase-based approach uses a combination of band pass and lowpass filters instead of just lowpass filters.

Phase-based video motion magnification allows separating the amplitude and phase on each spatial scale and orientation of the pyramid, once the intensity of the frames has been decomposed into Fourier series, assuming that the video is mostly static. According to the Fourier shift theorem it is possible to relate a phase shift in the frequency domain with a translation into the spatial domain, so it is possible to associate phase differences with motion.

The local phases over time at every spatial scale and orientation of the Steerable pyramid are computed for all the frames, and their differences with the corresponding values for the first frame calculated. Then, a temporal bandpass filter is applied to those differences in order to select the frequency range of interest and remove any temporal DC component (the average luminance of the frame). Then, as in EVLM, a magnification parameter is applied to the phase shifts, which allows to make small movements of the objects visible. Finally, the pyramids containing the amplified signals are reconstructed and added back to the original input to construct the magnified video.

For the specific application proposed in this work, the detection of breathing movements, most of the videos have oscillatory movements without a long duration and small amplitudes. However, this method can be used to amplify non-periodic motions as well, as long as they are within the passband of the temporal filter.

#### Steerable pyramids creation: Spatial filtering over the frames

As mentioned before, the first step in PBVM is the computation of a 2D Discrete Fourier Transform (DFT) over all frames in the video, and the subsequent application of the spatial filters, with different size and orientation. This produces a linear multi-scale and multi-orientation image decomposition named Steerable pyramid [19–22]. Each level of the pyramid is an array of complex numbers, and their phase and amplitude are computed, element by element.

Following the notation in Eq (1), the signal *I*(*x* + *δ*(*t*)) is decomposed into a Fourier series

$$\begin{array}{c} I\left(x+\delta \left(t\right)\right)=\sum_{\omega =-\infty }^{\infty }A_{\omega }e^{i\omega (x+\delta (t))} \end{array}$$ (3)

in which each single frequency *ω* corresponds to each band of the pyramid

$$\begin{array}{c} S_{\omega }\left(x,t\right)=A_{\omega }e^{i\omega (x+\delta (t))} \end{array}$$ (4)

where the phase *ω*(*x* + *δ*(*t*)) contains motion information.

**Fig 4:**
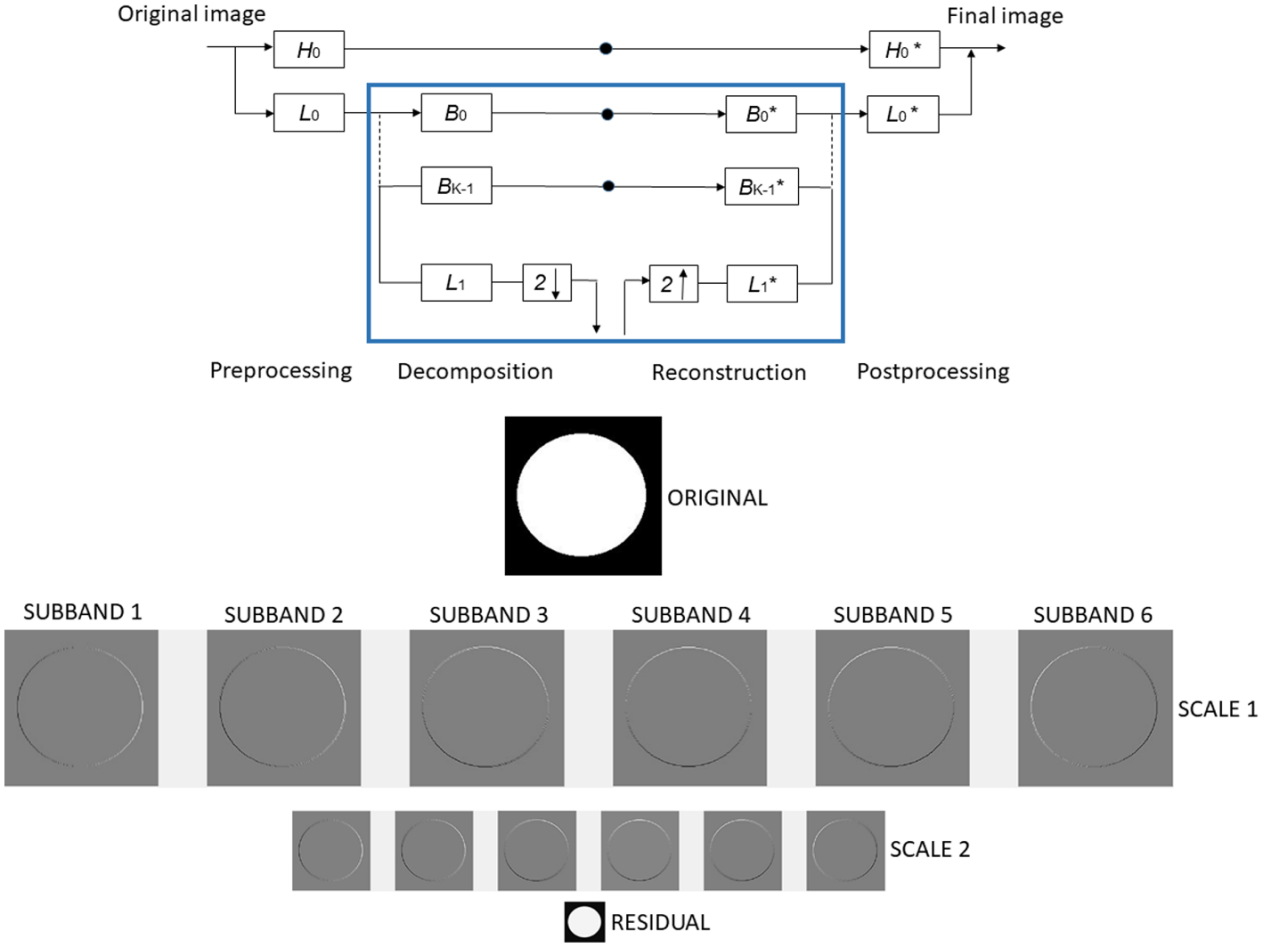
At the top of the image a scheme of the Steerable pyramid construction is shown. A particular application is shown at the bottom, where each row represents a scale and each column a band. The original image (top), the oriented bands at each scale (middle) and the lowpass residual (down), are shown.

The procedure to create a Steerable pyramid is shown in Fig 4, where H, L and B, represent high, low and bandpass filters, respectively. The recursive construction/reconstruction of the pyramid is carried out by repeating the highlighted rectangle in blue. Boxes containing “2 ↓” and “2 ↑” correspond to downsampling and upsampling by a factor of 2, respectively. The asterisks indicate a 180° rotation of the filters. At the bottom of Fig 4 an example of a Steerable pyramid with 6 orientation bands (bandpassed images) and 2 scales, using the image of a white circle on a black background, is shown. The steerable filters produce rotations that can be appreciated by looking at the white part of the circle contour.

#### Temporal filtering and magnification

Once the pyramid has been built, the phase on each spatial scale and orientation separated, and their differences computed, a temporal bandpass filtering is applied, looking for the isolation of the motion in the specific temporal frequencies to be magnified. So, if the phase in (3) is *ω*(*x* + *δ*(*t*)), after applying the mentioned bandpass filter

$$\begin{array}{c} B_{\omega }\left(x,t\right)=\omega \delta \left(t\right) \end{array}$$

is obtained.

Once the temporary filter has been applied, the result is multiplied by an amplification factor *α*, obtaining the motion magnified band:

$$\begin{array}{c} {\hat{S}}_{\omega }\left(x,t\right)=S_{\omega }\left(x,t\right)e^{i\alpha B_{\omega }}=A_{\omega }e^{i\omega (x+(1+\alpha )\delta (t))} \end{array}$$ (5)

The result ${\hat{S}}_{\omega }\left(x,t\right)$ is a complex sinusoid whose movement is (1 + *α*) times higher than the original.

Changing the *α* value, it is possible to make perceptible different movements in the input video, although there are some limitations in the degree of magnification. As *α* increases, the output video has more noise and artifacts. In fact, as with the ELVM, there is an upper limit for *α* when an octave-bandwidth (4 orientations) Steerable pyramid is used (see [17]):

$$\begin{array}{c} \alpha \delta \left(t\right)<\frac{1}{4\omega } \end{array}$$ (6)

When a half-octave (8 orientations) Steerable pyramid instead of a octave-bandwidth one is used, the threshold for *α* is given by:

$$\begin{array}{c} \alpha \delta \left(t\right)<\frac{1}{2\omega } \end{array}$$ (7)

According to Eqs (6) and (7), it seems clear that motions with low spatial frequencies can be magnified more than those with high spatial frequencies.

#### Video reconstruction

The final step of the video magnification procedure consists of reconstructing the video from the *α* times magnified pyramid levels. This is performed by the inverse process of the steerable pyramid creation. In practice all the operations are performed as component-wise multiplications in the Fourier domain.

Once all levels of each frame have been reconstructed, they are summed. Subsequently, the lowpass residuals of each frame that has the lowest resolution (obtained applying the spatial filter with the smallest dimensions to the original frame)are added. Finally, adding this result to the original signal, the output video is obtained. It should bee taking into account that throughout the process several bandpass filters have been applied, consequently, it is necessary to add a high-pass residue image to reconstruct the final image.

## Simulating a scenario with injured people

In order to verify that the proposed methodology works properly, an accident such as a gas leak or a terrorist attack, with three injured people involved, was simulated. Victims are unconscious at first glance, and the video magnification procedure explained before was applied to make small movements produced by breathing perceptible. The three people who appear in the video are co-authors of the article, and all of them have given their verbal consent to carry out the simulation. They are all professors at the University of Oviedo, from where they were recruited at a meeting held on May 2, 2017. The simulation was submitted to the Ethical Committee of the Principality of Asturias (CEICR), which evaluated it and provided the appropriate authorization to carry it out.

A video camera (Panasonic Lumix DMC-TZ80) which has a 30x optical zoom and HD resolution, mounted on a tripod located several meters away from the people, was used. After setting the camera on movie mode, volunteers were asked to lie down in the three different positions in which a victim can be normally found: lying on their back, face-down and sideways.

Movies were recorded filming the whole scene without additional optical lenses. The length of the video was long enough to capture the movement to be magnified. Normally a video of a couple of minutes or even less was sufficient. Daylight was used as the illumination source in combination with normal artificial fluorescent light to improve the image sharpness. The movie was recorded in color with 50 frames per second (fps) and 1920 × 1080 pixel resolution. As an adult takes 12-18 breaths per minute or 0.2-0.3 breaths per second, the sampling rate requirements are fulfilled. It was saved in MP4 format and transferred to a PC to be processed using Matlab R2015b software.

**Fig 5:**
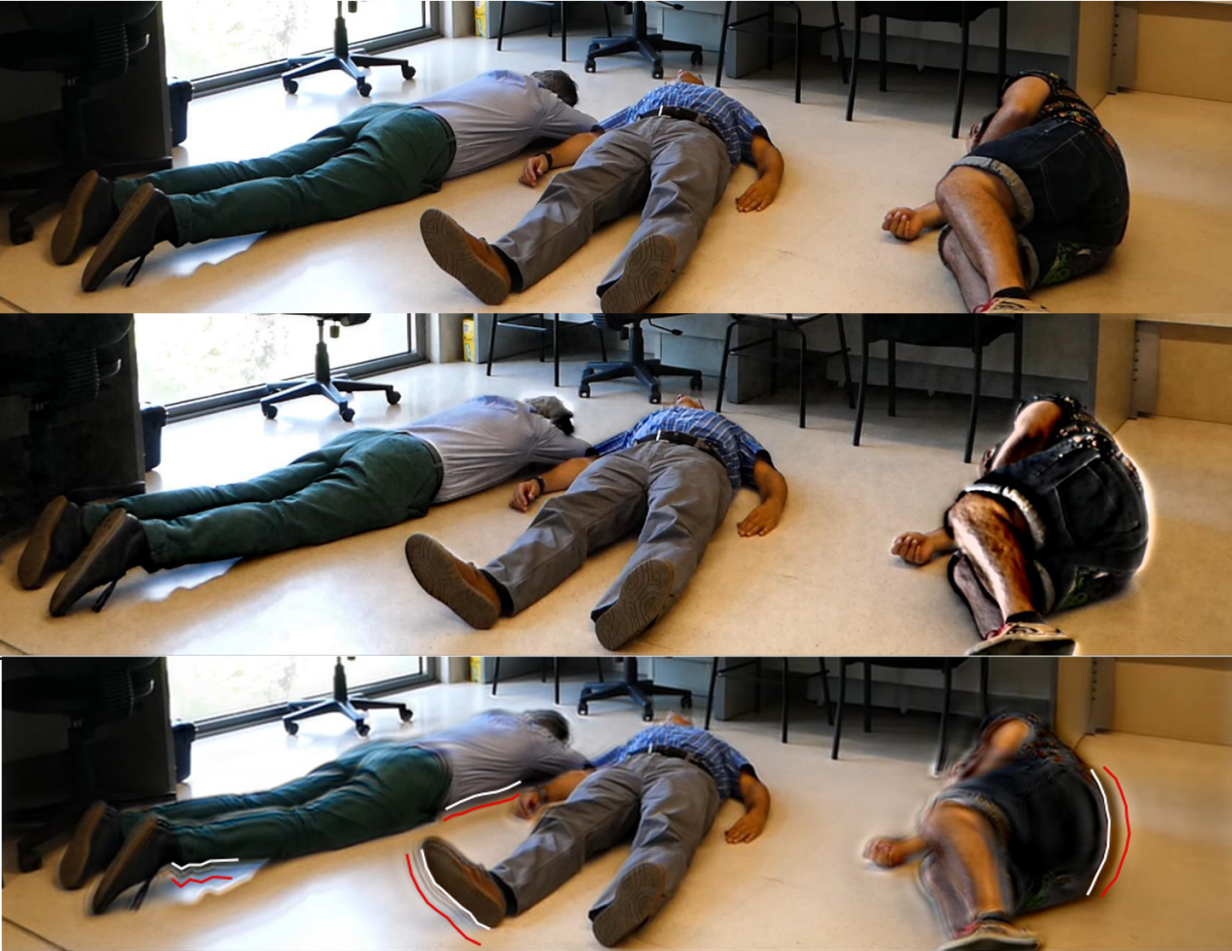
From top to bottom: Original video, a frame of the magnified video using the ELVM, another frame of magnified video using PBVM algorithm. Original and magnified silhouettes using PBVM algorithm are highlighted in white and red, respectively (*α* = 50 on both cases).

After performing many tests with values of the magnification factor between 5 and 60 in increments of 5, it was found that ELVM with small values of the magnification factor produces an almost imperceptible magnification, and that the quality of the image obtained by this algorithm gets worse as *α* increased. In order to increase the magnification without increasing the noise, the PBVM algorithm was tested. Visually, the best result was obtained for a magnification factor of around *α* = 50. For those values satisfying *α* ≤ 10, the motion magnification was almost imperceptible, and for *α* ≥ 50 motion magnification was too big, originating a blurry video. The difference between the results using both procedures, with the same amplification factor *α* = 50, is shown in Fig 5, where some segments of the silhouette of the people, both in a frame of the original video and in the magnified frame, were highlighted. This procedure allows to have a quick overview of the utility of video magnification to detect movements due to breathing.

Although in the ELVM approach the magnification was not large enough to be appreciated in Fig 5, the fact is that it is quite evident in the magnified video. However, a great magnification is obtained for the same value of *α* using PBVM method, although the edges of the moving bodies appear somewhat distorted. Nevertheless, color changes of the entire image and noise are almost imperceptible, and no significant noise is added to the scene. The original video, and the two magnified videos corresponding to Fig 5, are available in the supplementary material (available at https://figshare.com/articles/Video_magnification_to_detect_human_vital_signs/5769840).

**Fig 6:**
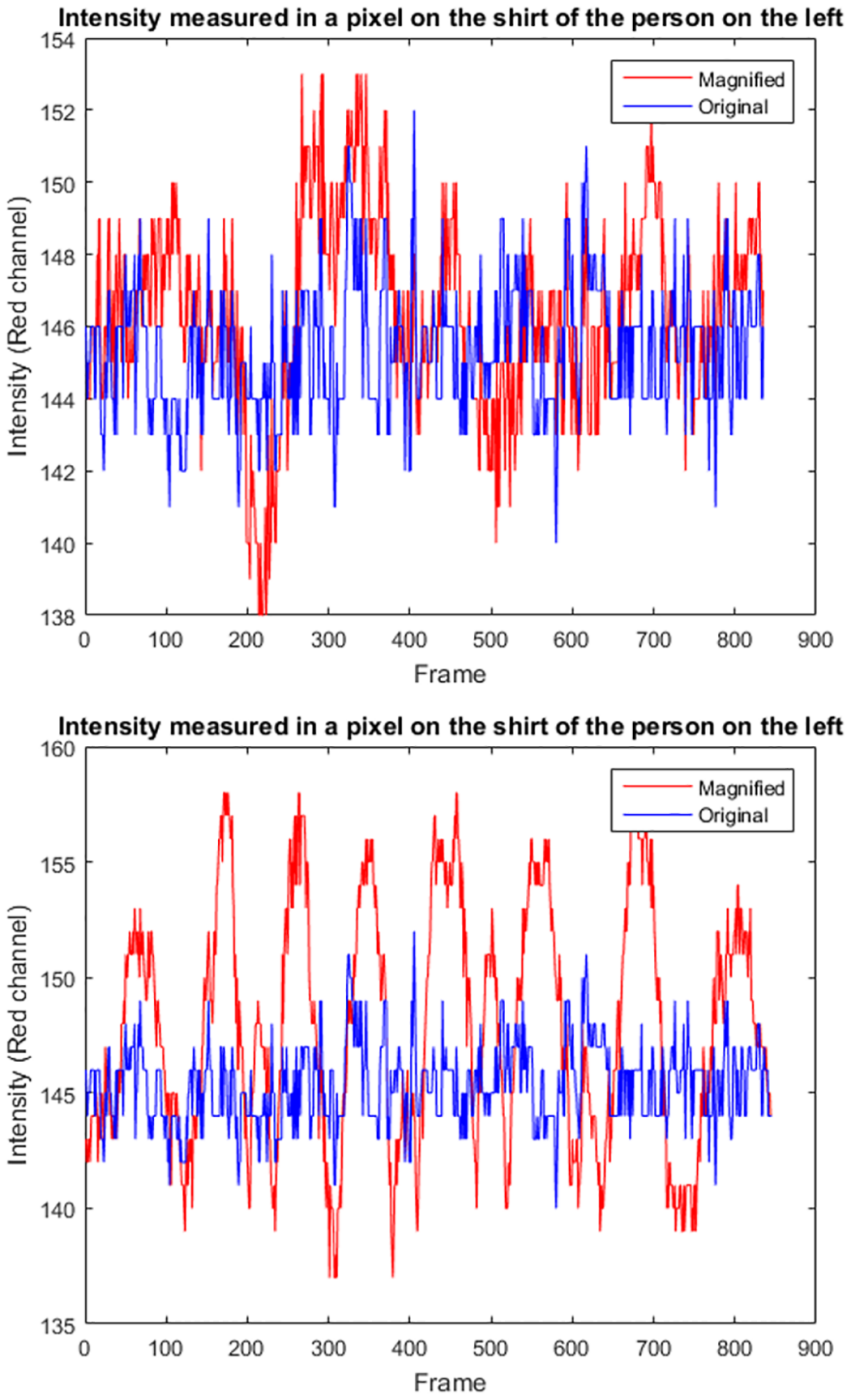
Comparison of the intensity of a pixel (in the red channel) in the original and in the output video obtained by ELVM (up) and by PBVM algorithm (down), respectively.

In order to quantify the capacity of both approaches, ELVM and PBVM, to increase the intensity signal, a set of pixels of a region where the movement is more evident (for instance, a pixel of the shirt of the person on the left) were selected, and the intensity in the original video, as well as in the output magnified videos, were obtained. Results are shown in Fig 6 for a pixel, where it is noticeable that the increase of the pixel intensity is significantly greater using PBVM magnification. The same pattern was found for other pixels in the video.

The increasing of the pixel intensity due to magnification is normally associated with a decrease of the quality of the video. To check this, the quality of both output videos by means of the Peak Signal to Noise Ratio (PSNR) was estimated:

$$\begin{array}{c} PSNR=10\cdot log(\frac{255^{2}}{MSE(I_{O},I_{M})}) \end{array}$$ (8)

where *I*_*O*_ and *I*_*M*_ are the intensities in the original and in the final video, respectively; MSE being the mean square error, given by:

$$\begin{array}{c} MSE\left(I_{O},I_{M}\right)=\frac{1}{MN}\sum_{y=1}^{M}\sum_{x=1}^{N}{\left(I_{O}-I_{M}\right)}^{2} \end{array}$$ (9)

For an image in RGB format, the MSE is calculated as the arithmetic mean of the MSEs for each red, blue and green color component.

**Fig 7:**
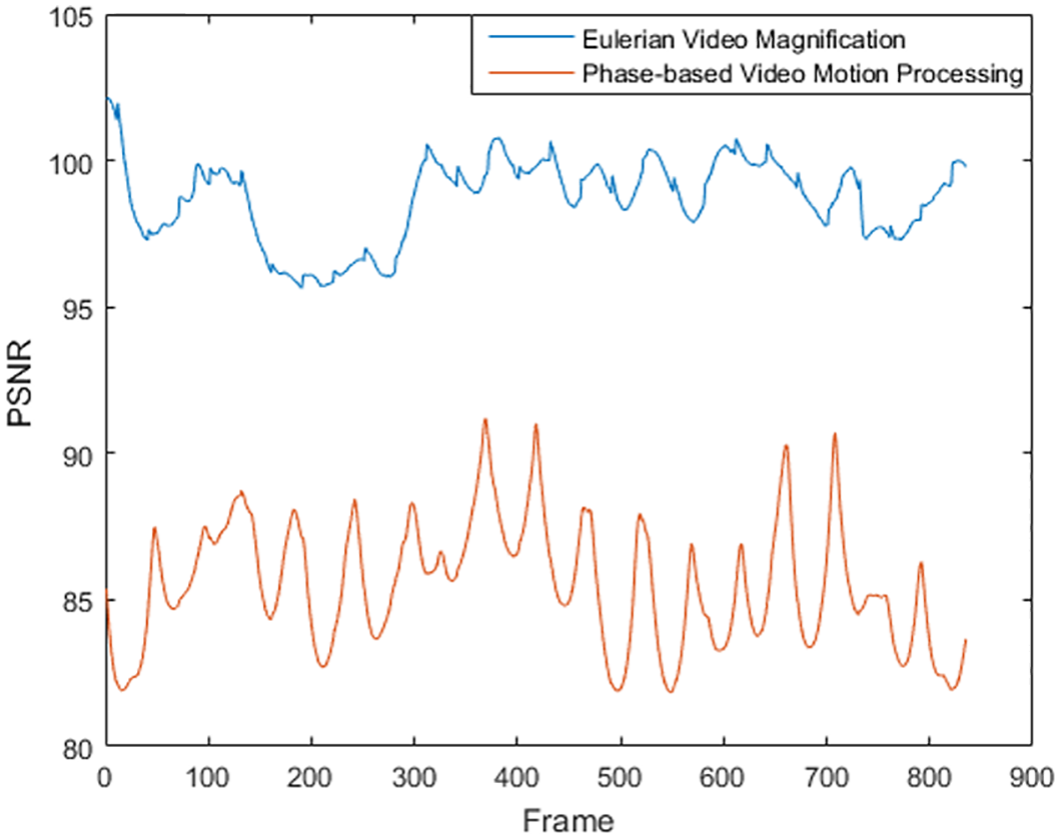
PSNR of the magnified video obtained after applying both algorithms to the same input video and magnification parameter (*α* = 50).

The PSNR is dimensionless, as both numerator and denominator represent pixel values. However, it is usually expressed in decibels (dB), although it is less sensitive to changes in the MSE. As it has no absolute meaning, it is meaningless to say that a determined value of PSNR is good or not. It is only a way of comparing the effects of the *α* value on the performance of an algorithm. Fig 7 shows the PSNR obtained for both magnification methods. It is clear from this figure that, effectively, the EVLM algorithm produces an image of higher quality. However, as was previously mentioned, the magnification is better appreciated in the phase-based magnified video.

## Discussion

The simulation explained in the previous section was repeated in different situations concerning light conditions in the room, distance to the objects, camera movement and camera frame rate, in order to determine the best for video magnification. Below, is discussed how each of these aspects affects the results of video magnification.

Since the main idea of this article is to magnify small movements that are not visible to the naked eye, it is crucial to ensure that the camera is not subject to any vibration or sudden movement, such as those produced by pressing the record button. If these movements have a frequency within the range that the user has chosen, they will also be magnified. Moreover, camera displacements modifying the framing result in unacceptable magnified videos. Therefore, it is very important to place the camera on a stable element, preventing inappropriate movements at the time of video recording. In this sense, it is advisable to operate the camera remotely. In fact, an experiment was conducted filming with a camera mounted on a UAV (unmmanned aerial vehicle), and even though the video was fairly stable it was not possible to magnify the motion due to breathing without magnifying the small movements of the camera. The results did not improve even using a video stabilization algorithm. As a result, the magnified videos showed distorted frames that do not allow to perceive breathing movements.

Similarly, lighting conditions is another factor to be considered. Suitable conditions allows to obtain images with greater sharpness. For this reason, it is advisable to record the video in good environmental conditions, minimizing the existence of shadows. However, movements due to breathing were also detected in scenarios with poor lighting conditions. This suggest that the propposed method is valid in many real situations.

Regarding the distance of the camera to the objects, it is advisable to locate the camera close enough to allow detecting the victims. For a camera with HD image resolution, the camera can be placed up to several tens of meters away. Obviously, this distance increases with the video resolution. In our experiment, distances from 3 to 10 meters were tested, and it was possible to detect breathing movements even at the largest distance.

Regarding the speed of video recording, it should be enough to record the frequencies of vibration according to the Nyquist criterion. Then, low-cost video cameras of 20 fps are suitable for our particular problem.

Concerning the magnification factor, although it is a critical aspect to take into consideration, it is also true that it may vary over a fairly wide range of values without producing noticeable effects. Our recommendation is to try several values in steps of 5 to 10 units.

According to the tests performed, it can be said that the average value of the PSNR is higher using the intensity linearization procedure than the phase-based procedure. This means that the first method produces an output magnified video with less noise. However, this is only valid for small magnification factors, since the quality of the output magnified video decreases with the value of the magnification.

In contrast, attending to the processing time, the phase-based method is considerably slower than that based on intensity linearization. Therefore, if obtainig results in almost real time is not priority, the phase-based method should be employed. Otherwise, it is better to employ the linearization method, although videos of worse quality will be obtained using the same magnification, making more difficult to identify signs of life. In this context, ELVM provided adequate results in just a couple of minutes when the original video was resampled to half the original cell size. This time could be reduced if the frames are cropped to select just the area containing the people under study, since this reduces the image extension. On the other hand, the PBVM algorithm took about 15 minutes in the same conditions, although, as mentioned, the magnified video showed the movement due to breathing much more clearly.

## Conclusion

In a standard video, hidden signals with small amplitude may exist. Often, these signals cannot be detected with the naked eye because of its limited sensitivity. We propose to use video magnification techniques to detect slow body movements due to breathing, as a method to identify injured people in accident or violent scenarios. Two different video magnification procedures were tested in a simulated scenario with injured people. Both methods, one based on a linear decomposition of the pixel intensity, and the other based on amplifying phased variations of the coefficients of a Steerable pyramid, proved to be useful. However, although the first method is simple and fast, the noise increases linearly with the amplification factor, so it only supports small values of this factor. The second methods provided the best results in terms of magnification because it supports a larger amplification factor, although it requires a considerably longer runtime.

Obviously, the processing time depends on the length of the video, which must be long enough to capture a representative sample of the movement to be magnified, showing phenomena occurring at the temporal frequencies selected by the user. In addition, the global processing time of a video increases with the number of levels that form the pyramid, but more decomposition means less noise in the output video.

Among the different factors concerning the utility of the video magnification, a fundamental aspect to be considered at the time of recording the video is that the camera has to remain stable during data collection. In addition, light conditions are also a factor to be considered when striving for good results.
